# Preserved retinal sensitivity in spatial correspondence to an intrachoroidal cavitation area with full thickness retinal defect: a case report

**DOI:** 10.1186/s12886-016-0362-6

**Published:** 2016-10-26

**Authors:** Yoshiyuki Kita, Makoto Inoue, Gábor Hollό, Ritsuko Kita, Masahiko Sano, Akito Hirakata

**Affiliations:** 1 Department of Ophthalmology, Kyorin University School of Medicine, 6-20-2 Shinkawa, Mitaka, Tokyo, 181-8611 Japan; 2 Department of Ophthalmology, Semmelweis University, Budapest, Hungary

**Keywords:** Glaucoma, High myopia, Intrachoroidal cavitation, Perimetry, Retinal defect, Retinal nerve fiber layer, Visual field

## Abstract

**Background:**

Intrachoroidal cavitation (ICC) in the temporal peripapillary area is a relatively frequent finding in high myopia. However, ICC associated with a full thickness retinal defect rarely observed. We report an unusual case of ICC combined with a full thickness retinal defect in the papillo-macular bundle, in which the spatially corresponding visual field sensitivity was preserved.

**Case presentation:**

A high myopic and pseudophakic left eye of a 79-year-old Japanese woman was evaluated with swept source optical coherence tomography (SS-OCT) and Humphrey 30-2 visual field testing for moderate glaucoma. The best-corrected visual acuity was 20/20, the axial length was 28.77 mm, the mean deviation -8.94 dB, and the intraocular pressure was 15 mmHg without medication. The horizontal SS-OCT scans showed a wide ICC with a full thickness retinal defect in the papillo-macular area at the outer border of the myopic peripapillary beta zone atrophy. The retina was herniated into the ICC area. While no sensitivity loss was seen in the central visual field corresponding to the full thickness retinal defect, a glaucomatous visual field deterioration spatially corresponding to the glaucomatous disc damage was present. The preserved retinal sensitivity spatially corresponding to the full thickness retinal defect was confirmed with microperimetry.

**Conclusions:**

Our case suggests that retina herniated in peripapillary ICC temporal to the disc may preserve some function despite the presence of a retinal defect.

## Background

On retinal photography and with clinical evaluation of the retina peripapillary intrachoroidal cavitation (ICC) typically appears as an area surrounded by a yellowish–orange lesion that is located inferior or temporal to the optic disc in highly myopic eyes [1, 2]. Several case reports were published on ICC [1–5], and there have been reports on ICC case associated with a full thickness retinal defect [3, 5]. It has been shown that a glaucomatous visual field defect is also typically seen in ICC, although a causal relationship between ICC (with or without a retinal defect) and the visual field sensitivity loss has not been established [3]. We report on an unusual case of temporal peripapillary ICC associated with retinal herniation and full thickness retinal defect with preserved retinal sensitivity in the corresponding central visual field.

## Case presentation

**Fig. 1:**
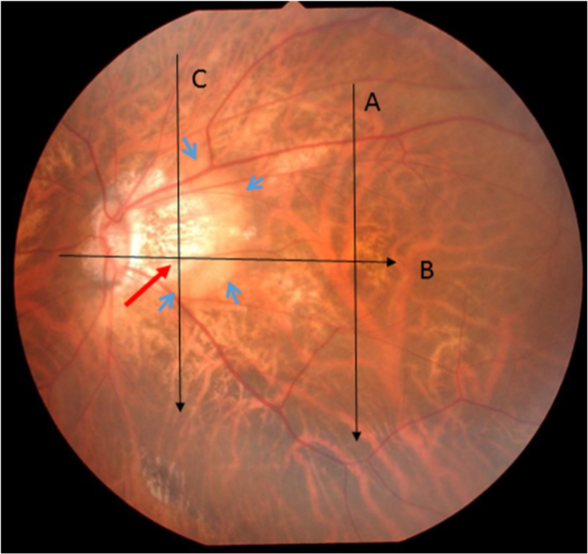
Fundus photograph of the left eye. Glaucomatous optic neuropathy, a large temporal beta zone peripapillary atrophy and a *yellowish–orange* lesion located temporal to the optic disc (*blue arrows*) are present. Swept-source optical coherence tomography scan directions for Fig. 3a, b and c are shown as *black lines with arrows*. The retinal defect is in the peripapillary atrophy (*red arrow*)

**Fig. 2:**
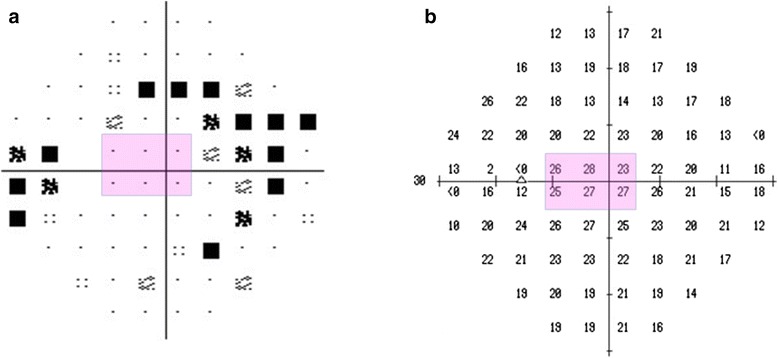
Visual field of the left eye. Pattern deviation plot (**a**) and threshold plot (**b**) of the central 30° visual field (Humphrey Field Analyzer, Swedish Interactive Threshold Algorithm) show superior and inferior Bjerrum scotomas corresponding to the superior and inferior neuroretinal rim loss, respectively. The retinal sensitivity is preserved in the papillo-macular bundle area (pink area) which spatially corresponds with the full thickness retinal defect

**Fig. 3:**
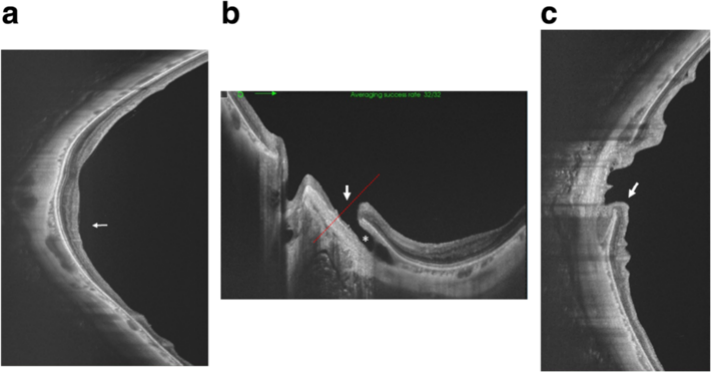
Optical coherence tomography (OCT) image of the posterior pole of the left eye. **a** Vertical swept source (SS) -OCT B scan through the fovea. Localized retinal nerve fiber layer thinning is particularly evident in the inferior area (*white arrow*). **b** Horizontal SS-OCT scan shows an intrachoroidal cavitation area (white asterisk) temporal to the optic nerve, and a full thickness retinal defect (*white arrow*) along the temporal border of the myopic peripapillary atrophy. The *red dotted line* shows the scanned area of (**c**). **c** Vertical OCT scan shows herniation of the retina toward the intrachoridal cavitation (*white arrow*)

**Fig. 4:**
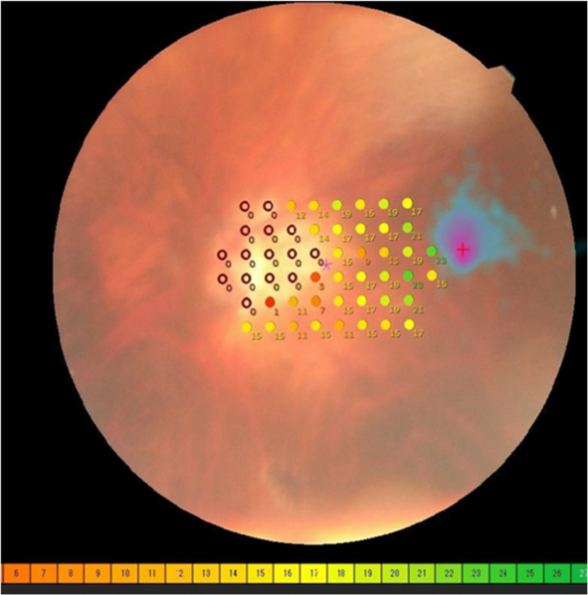
MP-3 microperimetry results. The sensitivity of the papillomacular bundle was tested with a customised 52 points program using “*white*” test lights (stimulus size Goldmann III, duration 200 ms) presented on a dim “*white*” background using a 4-2 threshold strategy. MP-3 microperimetry showed an absolute scotoma spatially corresponding to the optic disc and the peripapillary beta zone area *(purple circles*, 0 dB). In contrast, no scotoma was found in the papillo-macular bundle, which spatially corresponds to the full thickness retinal defect

A high myopic and pseudophakic 78-year-old Japanese woman was referred to us for assessment of her moderate glaucoma of the left eye. The right eye previously underwent an unsuccessful pars plana vitrectomy for macular hole. On the left eye best-corrected visual acuity was 20/20, the refractive error -3.00 diopters, the axial length 28.93 mm, and the intraocular pressure 15 mmHg with no medication. The optic nerve head showed a moderate superior and a severe inferior glaucomatous neuroretinal rim loss with a laminar dot sign (Fig. 1). The central 30° visual field evaluated with the Humphrey Field Analyzer, 30-2 Swedish Interactive Threshold Algorithm standard program (Carl Zeiss Meditec, Dublin, CA) was reliable and showed a mean deviation value of -8.94 dB and typical glaucomatous deterioration (superior and inferior Bjerrum scotomas) spatially corresponding to the disc damage (Fig. 2). In addition to the above findings on clinical examination a yellowish–orange lesion was seen in the horizontal meridian temporal to the optic disc (Fig. 1, asterisk). Swept source optical coherence tomography (SS-OCT; DRI OCT-1 Atlantis, Topcon, Tokyo, Japan) was performed. In the suspected location the horizontal OCT scans showed a wide ICC with a full thickness retinal defect at the outer border of the myopic peripapillary beta zone atrophy, and herniation of the retina in the ICC area (Fig. 3). Retinal nerve fiber layer thinning in the superior and inferior hemisphere areas were also found with SS-OCT (Fig. 3). Since no sensitivity loss was seen in the central visual field spatially corresponding to the position of the full thickness retinal defect [6] (Fig. 2), microperimetry with the MP-3 system (Nidek, Aichi, Japan) was performed. Microperimetry revealed an absolute scotoma spatially corresponding with the peripapillary atrophy but showed no scotoma in the area of the papillo-macular bundle, where the full thickness retinal defect was found (Fig. 4).

## Discussion and conclusions

ICC is common in pathological myopia and visual field defects are typical in eyes with ICC. However, association of ICC and a full thickness retinal defect in the area of the papillo-macular nerve fiber bundle has not yet been published. In one investigation 71 % of eyes with ICC showed a glaucomatous visual field defect [3]. Ohno-Matsui et al. [1] reviewed the medical records of 125 pathological myopia patients whose papillary and peripapillary regions were examined with SS-OCT. Sixteen of the 125 patients (12.8 %) were found to have a temporal ICC. In addition, an inner retinal defect was observed in 2 of the 16 patients. Though, our case is typical in terms of clinical characteristics (high myopia in the background; ICC location temporal to the disc; and typical clinical appearance of the ICC) it is unusual due to the association of ICC and a full thickness retinal defect in the papillo-macular bundle area; and the lack of association of the full thickness retinal defect with any spatially corresponding functional loss in the central visual field and in microperimetry of the macula. Since preservation of retinal function was confirmed with two different functional tests (perimetry and microperimetry); the visual field was reliable; and the coexisting glaucomatous visual field deterioration was correctly detected, we think that the preservation of visual function corresponding with the papillo-macular bundle is a valid finding. As far as we know this is the first report in which preserved retinal sensitivity, confirmed with two different methods is seen in ICC associated with a full thickness retinal defect. We suppose that the herniated retina maintained some of its function in the papillo-macular area despite the full thickness damage, which may suggest a relatively a recent development of the ICC. Since the time course of the development of functional deterioration due to ICC formation is unknown further prospective investigations are proposed to clarify the relationship between ICC formation and corresponding perimetric function.

## Abbreviations

ICC: Intrachoroidal cavitation
SS-OCT: Swept source optical coherence tomography
